# Dataset on records of *Hericium erinaceus* in Slovakia

**DOI:** 10.1016/j.dib.2017.02.056

**Published:** 2017-03-31

**Authors:** Vladimír Kunca, Marek Čiliak

**Affiliations:** Technical University in Zvolen, Faculty of Ecology and Environmental Sciences, Department of Applied Ecology, T.G. Masaryka 24, SK-960 53 Zvolen, Slovakia

## Abstract

The data presented in this article are related to the research article entitled “Habitat preferences of *Hericium erinaceus* in Slovakia” (Kunca and Čiliak, 2016) [FUNECO607] [2]. The dataset include all available and unpublished data from Slovakia, besides the records from the same tree or stem. We compiled a database of records of collections by processing data from herbaria, personal records and communication with mycological activists. Data on altitude, tree species, host tree vital status, host tree position and intensity of management of forest stands were evaluated in this study. All surveys were based on basidioma occurrence and some result from targeted searches.

**Specifications Table**

**Table:** 

Subject area	*Biology*
More specific subject area	*Mycology*
Type of data	*Table*
How data was acquired	*Survey, Field monitoring, Web source*
Data format	*Raw, Filtered*
Experimental factors	*All parameters of available data was taken and processed, forest naturalness was evaluated in-situ for 38 records*
Experimental features	*Monitoring of basidiomata, Literature retrieval, Herbaria retrieval, Field search, Getting information from mycological activists, Data analysis*
Data source location	*Slovakia*
Data accessibility	*Data is available with this article*

**Value of the data**

• Data on *Hericium erinaceus* basidiomata occurrence in nature are rare in almost the whole Europe
• Our data can contribute to better understanding of indicator value of the species
• List of available specimens can help in further research of the fungus (e.g. molecular analysis)

## 1 Data

In total, 60 records of *H. erinaceus* from Slovakia were included in this study, of which basidioma specimens exist or were confirmed in 19 records. A majority (70%) had accompanying altitude, all host tree identification (tree genus or species) and vital status and standing or fallen stem indication. We were able to assign forest stand naturalness to 38 of the 60 records.

## 2 Experimental design, materials and methods

We compiled a database of unpublished records of collections of *H. erinaceus* in Slovakia by processing data from herbaria (public herbaria BRA, SLO – abbreviations of public herbaria adhere to Index Herbariorum [3]), personal records (private herbarium of Vladimír Kunca (PVKU)) and communication with mycological activists (www.nahuby.sk). These records are summarized in Table 1. The records originate from the whole territory of Slovakia and include habitats considered suitable for the species. More than 20 individual collectors contributed most of the records collected from a range of forest types throughout the year.

Data on altitude, host tree species, host tree vital status, host tree position and intensity of management of forest stands were included in this study. All surveys were based on basidioma occurrence and some result from targeted searches. All records describing the same tree, stem or stump were evaluated only once.

Classification of forest stands in terms of management and naturalness followed that of [1]. Virgin forest is almost unaffected by humans, typical by natural tree species composition, spontaneous development, multi-aged structure, long continuity. Natural forest is formed by natural processes but influenced by human activities in the past (50 years and more ago, namely selective cutting of individual trees, selective removal of fallen or cut trees). In near-natural forest tree species composition largely corresponds to habitat conditions but the spatial structure is simpler than in the virgin or natural forest. Cultural forests are man-influenced forests where tree species composition corresponds more or less to habitat conditions but the stand is under permanent influence of conventional forest management. The species was also found in biotopes other than forest (in Table 1 in parenthesis) – in steppe (sandy soil with solitaire trees in region of very warm climate) and in city (solitaire tree in the urban ecosystem).

The categorisation of forest stands according to forest management plan (commercial, protection and special purpose forests) was considered top level, followed by in-situ assessment of trees species composition, age, horizontal and vertical structure, dead wood presence and occurrence of veteran trees. Actual tree species composition was compared to expected on the basis of forest site classification (based on soil, climate, water regime and landscape conditions) [4].

## Figures and Tables

**Table 1:** Unpublished records of *Hericium erinaceus* in Slovakia with available data and categorisation of forest stands in terms of management and naturalness.

No	Date	Locality	Host tree	Host tree vital status	Host tree position	Altitude (m)	Type of forest management	Source of information	Specimen in herbaria
1	27.8.2002	Hrochoť-ská dolina	*Quercus petraea*	dead	standing	753	virgin	Vladimír Kunca	
2	2.9.2005	nad Račou	*Quercus* sp.	dead	lying			František Jankovič	
3	6.11.2005	Považský Inovec	*Quercus* sp.	living	standing			Zuzana Štofková	
4	10.12.2005	Modra	broadleaf	living	standing			Soňa Jančovičová	SLO
5	7.10.2006	Zlatá Idka − Rudník	*Quercus* sp.	living	standing	437	natural	Vladimír Blaško	
6	26.9.2006	Jabloňové	*Fagus sylvatica*	dead	standing		natural	Pavol Ocelka	
7	22.10.2006	Priekopa	*Fagus sylvatica*	living	standing			Vladimír Macík	
8	21.11.2006	Jabloňové	*Fagus sylvatica*	living	standing			Pavol Ocelka	
9	4.1.2007	Machulin-ce	*Quercus* sp.	living	standing	345	natural	Michal Gašparík	
10	16.9.2007	Košická Nová Ves	*Quercus* sp.	living	standing		near-natural	Erik Brozmann	
11	15.9.2007	Jahodník	*Quercus* sp.	living	standing			Martin Felčír	
12	26.9.2007	Machulin-ce	*Quercus* sp.	living	standing	355	natural	Michal Gašparík	
13	18.8.2008	Jedľové Kostoľany	*Fagus sylvatica*	dead	lying			František Ježo	
14	14.9.2008	Pezinok	*Quercus* sp.	living	standing	342	cultural	Jaroslav Kuriplach	
15	30.9.2008	Pezinok	*Quercus* sp.	living	standing	337	cultural	Jaroslav Kuriplach	
16	25.8.2009	Prešov	*Fagus sylvatica*	living	standing			Martin Šandor	
17	7.10.2009	Pezinok	*Quercus* sp.	living	standing	322	cultural	Jaroslav Kuriplach	
18	11.10.2009	Modra	*Quercus* sp.	dead	stump			Bronislav Mandinec	
19	23.10.2009	Rača	*Quercus* sp.	living	standing			Matúš Hurbanič	
20	30.10.2009	Skalie	*Quercus petraea*	dead	lying	600	natural	Vladimír Kunca	PVKU
21	14.11.2009	Zobor	*Quercus* sp.	living	standing			Ivan Polák	
22	12.9.2010	Kuchyňa	*Fagus sylvatica*	living	standing	525	near-natural	Ladislav Špeta	
23	18.9.2010	Skalie	*Quercus petraea*	dead	standing	600		Vladimír Kunca	PVKU
24	1.10.2010	Senec	*Quercus* sp.	dead	stump	155		Ivona Kautmanová	BRA
25	3.10.2010	Zemplínska Šírava	*Quercus* sp.	dead	lying			Ivan Nagy	
26	4.10.2010	Zemplínske vrchy	*Quercus* sp.	dead	lying	300	cultural	Mikuláš Lazor	
27	5.10.2010	Priekopa	*Fagus sylvatica*	living	stump			Vladimír Macík	
28	11.10.2010	Topoľčany	*Fagus sylvatica*	dead	standing			Jozef Rešetka	
29	16.10.2010	Dúbravica	*Quercus* sp.	dead	lying	450	natural	Maroš Durdiak	
30	16.10.2010	Pezinok	*Quercus* sp.	dead	lying branch	353	cultural	Jaroslav Kuriplach	
31	12.11.2010	Zemplínske vrchy	*Quercus* sp.	living	standing			Mikuláš Lazor	
32	16.11.2010	Snina	*Fagus sylvatica*	dead	lying	440	cultural	Jozef Pavlík	PVKU
33	8.9.2011	Zemplínske vrchy	*Carpinus betulus*	dead	stump	300	cultural	Mikuláš Lazor	
34	7.10.2011	Hrochotský mlyn	*Quercus cerris*	living	standing	750	virgin	Vladimír Kunca	PVKU
35	9.10.2011	Pezinok	*Quercus* sp.	living	standing	394	cultural	Jaroslav Kuriplach	
36	19.10.2011	Skalie	*Quercus petraea*	living	standing	595		Vladimír Kunca	PVKU
37	1.11.2011	Dubová	*Fagus sylvatica*	living	standing	600	cultural	Pavol Kešeľák	
38	25.12.2011	Poštárka	*Quercus cerris*	living	standing	390	natural	Vladimír Kunca	PVKU
39	4.8.2012	Snina	*Fagus sylvatica*	dead	lying	445	cultural	Jozef Pavlík	PVKU
40	1.9.2012	Havešová	*Fagus sylvatica*	dead	lying	550	virgin	Matúš Škutka	
41	28.9.2012	Banský Studenec	*Quercus petraea*	dead	standing	590	natural	Vladimír Kunca	PVKU
42	14.10.2012	Majdán	*Quercus cerris*	living	standing	270	cultural	Dušan Solár	
43	20.10.2012	Čifáre	*Quercus cerris*	dead	lying	250	cultural	František Hubina	
44	3.11.2012	Prievidza	*Quercus* sp.	dead	lying	443	natural	Stanislav Jankech	
45	25.11.2012	NNR Boky	*Quercus cerris*	dead	lying	452	virgin	Vladimír Kunca	PVKU
46	27.11.2012	Bratislava	*Fagus sylvatica*	living	standing	425	(city)	Ľudmila Čillíková	PVKU
47	22.9.2013	Pezinok	*Quercus* sp.	living	standing	332	cultural	Ľubica Kuriplachová	
48	24.9.2013	Senec	*Quercus* sp.	living	standing	149	cultural	Kvetoslava Balážová	
49	1.10.2013	Boky	*Ulmus carpinifolia*	dead	lying	347	virgin	Vladimír Kunca	PVKU
50	8.10.2013	Dubová	*Fagus sylvatica*	living	standing	600	cultural	Pavol Kešeľák	PVKU
51	15.10.2013	Pustý hrad	*Quercus petraea*	dead	lying	339	natural	Vladimír Kunca	PVKU
52	15.10.2013	Trenčians-ke Teplice	*Fagus sylvatica*	dead	lying			Vladimír Juhás	
53	17.10.2013	Kostoľany	*Quercus* sp.	dead	lying	578	natural	Anton Pánis	PVKU
54	17.10.2013	Kostoľany	*Quercus cerris*	living	standing	578	natural	Anton Pánis	PVKU
55	23.10.2013	Lučenec	*Quercus cerris*	living	standing	256	cultural	Štefan Baláž	PVKU
56	27.10.2013	Častá	*Quercus* sp.	dead	standing	235	near-natural	Patrik Štibraný	
57	17.11.2013	Svätý Jur	*Quercus* sp.			300		Ivona Kautmanová	BRA
58	23.8.2014	Poltár	*Quercus* sp.	living	standing	227	cultural	Ján Hraško	
59	26.9.2014	Somotor	*Robinia pseudoacacia*	dead	stump	103	(steppe)	Mikuláš Lazor	
60	4.10.2014	Lakšárska Nová Ves	*Quercus* sp.	living	standing	222	cultural	Kvetoslava Balážová	
